# Limited Clinical Utility of Remote Ischemic Conditioning in Renal Transplantation: A Meta-Analysis of Randomized Controlled Trials

**DOI:** 10.1371/journal.pone.0170729

**Published:** 2017-01-27

**Authors:** Chang-Cheng Zhou, Yu-Zheng Ge, Wen-Tao Yao, Ran Wu, Hui Xin, Tian-Ze Lu, Ming-Hao Li, Kai-Wei Song, Min Wang, Yun-Peng Zhu, Meng Zhu, Li-Guo Geng, Xiao-Fei Gao, Liu-Hua Zhou, Sheng-Li Zhang, Jia-Geng Zhu, Rui-Peng Jia

**Affiliations:** 1 Center for Renal Transplantation, Nanjing First Hospital, Nanjing Medical University, Nanjing, Jiangsu, China; 2 Department of Urology, Nanjing First Hospital, Nanjing Medical University,Nanjing, Jiangsu, China; 3 Department of Epidemiology and Biostatistics, School of Public Health, Nanjing Medical University, Nanjing, Jiangsu, China; 4 Department of Cardiology, Nanjing First Hospital, Nanjing Medical University, Nanjing, Jiangsu, China; University of Toledo, UNITED STATES

## Abstract

**Objective:**

We conducted this meta-analysis of randomized controlled trials (RCTs) to investigate whether remote ischemic conditioning (RIC) could improve graft functions in kidney transplantation.

**Methods:**

PubMed, Web of Science, and Cochrane Library were comprehensively searched to identify all eligible studies by October 5, 2016. The treatment effects were examined with risk ratio (RR) and weighted mean difference with the corresponding 95% confidence intervals (CI). The statistical significance and heterogeneity were assessed with both Z-test and Q-test.

**Results:**

A total of six RCTs including 651 recipients, were eventually identified. Compared to the controls, RIC could reduce the incidence of delayed graft function (DGF) after kidney transplantation (random-effects model: RR = 0.89; fixed-effect model: RR = 0.84). However, the decrease did not reveal statistical significance. The subgroup analysis by RIC type demonstrated no significant difference among the three interventions in protecting renal allografts against DGF. Furthermore, no significant difference could be observed in the incidence of acute rejection, graft loss, 50% fall in serum creatinine, as well as the estimated glomerular filtration rate and hospital stay between the RIC and Control groups.

**Conclusions:**

This meta-analysis suggested that RIC might exert renoprotective functions in human kidney transplantation, and further well-designed RCTs with large sample size are warranted to assess its clinical efficacy.

## Introduction

Kidney transplantation is the best treatment of choice for patients with end-stage renal disease (ESRD) [1]. Over the past few decades, renal transplantation has processed from a risky experimental approach to a safe and life-saving treatment; however, the shortage of renal allografts limited the clinical application [2]. To expand the organ pool, cardiac death and expanded-criteria donors have been suggested, which could worsen renal ischemia reperfusion injury (IRI) [3, 4]. During the transplantation procedure, IRI is an inevitable event, which could contribute to increased risk of delayed graft function (DGF), primary nonfunction, acute rejection (AR), as well as chronic renal fibrosis and graft loss. To date, numerous efforts have been conducted to avoid or alleviate IRI in renal transplantation, with the aim to improve the short- and long-term outcomes of renal recipients [5–7].

Remote ischemic conditioning (RIC) is an emerging and promising approach to attenuate postoperative renal IRI. In this strategy, several cycles of intermittent nonlethal ischemia and reperfusion are applied to a remote organ or issue, which could render the kidney more resistant against subsequent lethal ischemia and reperfusion [8–10]. According to diverse applied time points, RIC can be categorized into three types: remote ischemic preconditioning (RIPrC, induced before target organ ischemia), remote ischemic perconditioning (RIPeC, induced during target organ ischemia but before reperfusion), remote ischemic postconditioning (RIPoC, induced at the initiation of reperfusion) [11–13]. Even though the exact mechanism underlying RIC remains undefined, RIC has been validated as a potent nephroprotective strategy in animal models [13, 14].

Due to its lower economic cost, practical feasibility, and non-invasion, RIC has been translated rapidly from pre-clinical studies to clinical trials in kidney transplantation [15], and six randomized controlled trials (RCTs) have been conducted to evaluate the clinical efficacy of RIC in renal allograft recipients [15–20]. However, due to the limited sample size, the results remain inconclusive. Hence, we conducted this meta-analysis by pooling data from all eligible RCTs to evaluate the therapeutic efficacy and safety of RIC in renal transplantation.

## Materials and Methods

### Identification of eligible studies

This meta-analysis was conducted and reported in accordance with the PRISMA (Preferred Reporting Items for Systematic reviews and Meta-Analyses) guidelines [21]. A comprehensive electronic search of PubMed, Web of Science, and Cochrane Library was performed to identify all eligible studies with the following keywords: ischemic preconditioning, ischemic postconditioning, ischemic conditioning, or ischemic perconditioning; and kidney transplant or renal transplant (Last update: October 5, 2016), and the full search strategy in PubMed was presented in **S1 Table**. Furthermore, a manual search was also conducted for additional records through screening the reference lists of reviews and retrieved articles. No restrictions were applied on language.

### Selection criteria

Studies identified from the before-mentioned databases (PubMed, Web of Science, and Cochrane Library) were assessed by two independent authors (Chang-Cheng Zhou and Yu-Zheng Ge) according to the following predesigned inclusion criteria: (1) study design as RCT; (2) renal transplant recipients administrated with one type of RIC (RIPrC, RIPeC, or RIPoC); (3) sufficient data such as DGF, AR, estimated glomerular filtration rate (eGFR), 50% fall in serum creatinine (SCr), graft loss, and hospital stay were provided to evaluate acute or long-term outcomes. We excluded retrospective analyses, case reports, meeting abstracts as well as trial protocol.

### Data extraction

Two investigators (Chang-Cheng Zhou and Wen-Tao Yao) extracted data from eligible studies independently by using a predesigned data-collection form. The primary endpoint was set as the incidence of DGF, while the secondary endpoint as AR, graft loss, 50% fall in SCr, eGFR (1–3 months and 12 months post operation), and hospital stay. Furthermore, the following information was extracted: last name of first author, year, country, the demographic characteristics of the patients, RIC type, RIC protocol, donor type and preoperative hemodialysis utilization. Discrepancies were addressed by discussing with a third investigator (Rui-Peng Jia), and complete consensus on each item was resolved eventually. All the raw data was presented in **S2 Table**.

### Statistical analysis

For dichotomous variables and continuous variables, treatment effects were assessed as risk ratio (RR) and weighted mean difference (WMD) with their corresponding 95% confidence interval (CI), respectively. Subgroup analyses based on RIC type were also conducted. The statistical significance with respect to RR and WMD was assessed with *Z* test, and *P* values of less than 0.05 were considered significant.

The heterogeneity between eligible studies was explored by Chi-square based Q-test, and the presence of heterogeneity was considered significant if *P* <0.05 [22]. Both fixed-effect model (Mantel-Haenszel method) and random-effects model (DerSimonian and Laird method) were utilized to pool the outcomes from different studies, and the results yielded under random-effects model were presented in the main text. The one-way sensitivity analyses were performed to authenticate the effect of individual study on pooled outcomes and to detect the reliability of results by deleting a single study every time [23]. The publication bias analyses with Begg’s funnel plot and Egger’s linear regression test were not performed, as only six studies were included in this meta-analysis [24]. The sample size was calculated according to the DGF rate in RIC and control groups with a power of 0.8 (α = 0.05, β = 0.20, two-sided tailed) by using an embedded computing module in TSA software.

All statistical analyses were conducted with Review Manager (version 5.3, Cochrane Collaboration, Oxford, UK), STATA software (version 10.0; Stata Corporation, College Station, Texas, USA), and TSA software (version 0.9 beta, Copenhagen Trial Unit, Copenhagen, Denmark)

## Results

### Search results and study characteristics

**Fig 1:**
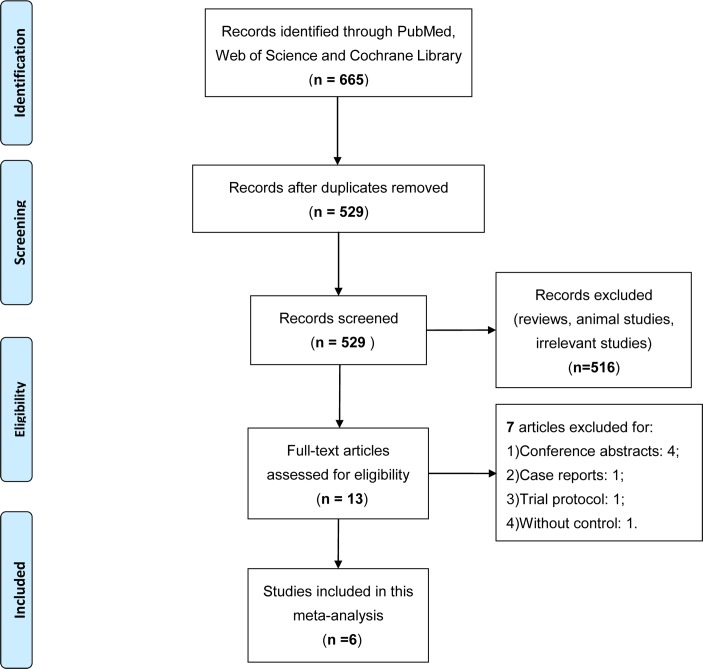
Flow diagram of study selection. Description: a total of six studies were included in this meta-analysis after a comprehensive study selection.

The search strategy initially generated a total of 665 records, among which 136 duplicates and 516 clearly irrelevant articles were excluded through reading title and abstract. After full-text assessment of the remaining 13 potentially relevant studies, seven were removed for: 1) conference abstracts; 2) case report; 3) trial protocol; and 4) without control. Finally, a total of six articles were eligible for the current meta-analysis [15–20], and the detailed screening process was presented in **Fig 1**.

A total of 651 renal transplant recipients were enrolled in the six studies, of which 325 recipients were randomly assigned to the RIC group, and the remaining 326 to the control group. Among those studies, two RCTs applied RIPrC [15, 17], one RIPoC [19], and remaining three RIPeC [16, 18, 20]. No significant difference was observed in the distribution of age, gender, and preoperative hemodialysis utilization between groups in each individual study, and the detailed characteristics of included studies were shown in **Table 1**.

**Table 1:** Characteristics of included trials.

Author	Year	No. of patients	Age (y)	Males (%)	RIC type	RIC procedure	Donor type	Preoperative HD n(%)
Krogstrup	2016	109/113	58.1(49.5–65.0)/ 61.4(49.4–66.6)	60/61	perconditioning	4 cycles of 5-minute ischemia and 5-minute reperfusion of the thigh	DCD/DBD	68(62)/65(58)
Nicholson	2015	40/40	45±14/47±14	67.5/52.5	perconditioning	4 cycles of 5-minute ischemia and 5-minute reperfusion of the thigh	Living-donor	23(58) / 20(50)
MacAllister	2015	102/99	47.6±15.1/46.8±15.1	72.4/61.1	preconditioning	4 cycles of 5-minute ischemia and 5-minute reperfusion of the arm	Living-donor	51(50) / 51(52)
Wu	2014	24/24	40.6±11.6/39.7±10.2	50/62.5	perconditioning	3 cycles of 5-minute ischemia and 5-minute reperfusion of the iliac artery	DCD	18(75) / 17(71)
Kim	2014	30/30	49(39–52)/46(36–50)	66.7/70	postconditioning	3 cycles of 5-minute ischemia and 5-minute reperfusion of the arm	Living-donor	27(90) / 28(93)
Chen	2013	20/20	30.6±7.0/32.5±10.3	70/80	preconditioning	3 cycles of 5-minute ischemia and 5-minute reperfusion of the thigh	Living-donor	Unclear

DCD: donation after cardiac death; DBD: donation after brain death; HD: hemodialysis; RIC: remote ischemic conditioning.

### Quality assessment

The quality of each individual trial was evaluated independently by two authors (Chang-Cheng Zhou and Ran Wu) using the Jadad score [25]. Among all six trials, four met high methodological quality and allocation concealment criteria [16, 17, 19, 20], and intention-to-treat analyses were apparently mentioned in three trials [16, 17, 20]. The detailed quality assessments were summarized in S3 **Table**.

### Study outcomes

#### Incidence of DGF

**Fig 2:**
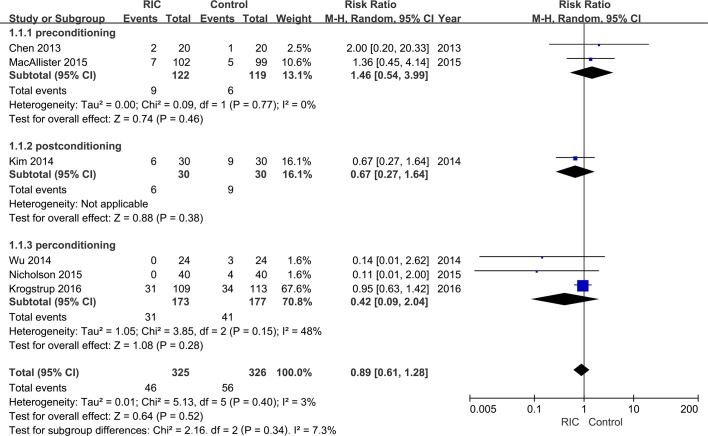
Forest plot with 95% confidence interval in DGF rates (random-effects model). Stratification analysis was conducted based on RIC types (RIPrC, RIPoC, and RIPeC).

After pooling data from all six trials, a trend of decline in the risk of DGF could be observed in the RIC group than the control group both under random-effects (RR = 0.89; **Fig 2**) and fixed-effect models (RR = 0.84; **S1 Fig**). However, unfortunately, the decrease did not demonstrate statistical significance under both models(random-effects model: 95% CI, 0.61–1.28, *P* = 0.52; fixed effect model: 95% CI, 0.60–1.18, *P* = 0.32;). Furthermore, the stratified analysis based on RIC type showed that all three interventions could not significant reduce the incidence of DGF (**Fig 2 and S1 Fig**).

#### Incidence of AR, graft loss, 50% fall in SCr

**Fig 3:**
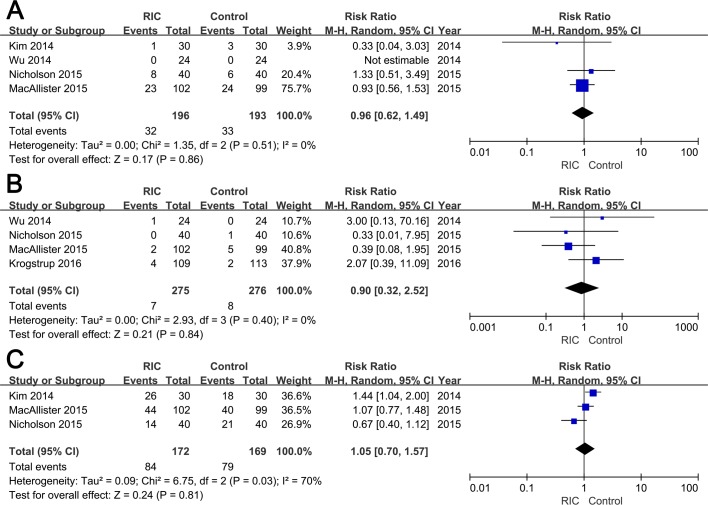
Forest plot with 95% confidence interval for dichotomous secondary end points (random-effects model). The incidence of AR (A), graft loss (B), and 50% fall in serum creatinine (C) in recipients treated with RIC compared with controls.

Four trials evaluated AR post transplantation [16–19], while in the study reported by Wu and colleagues, no recipients suffered AR during one-year follow-up [18]. No significant difference was detected in the incidence of AR between two groups (RIC vs. Control groups: RR = 0.96; 95% CI, 0.62–1.49; *P* = 0.86; **Fig 3A**). The incidence of graft loss within three months was documented in four studies [16–18, 20], and no significant difference was observed between groups (RIC vs. Control groups: RR = 0.90; 95% CI, 0.32–2.52; *P* = 0.84; **Fig 3B**). With regard to the rate of 50% fall in SCr within 24h post-transplantation, no statistical significant data (RIC vs. Control groups: RR = 1.05; 95% CI, 0.70–1.57; *P* = 0.81; **Fig 3C**) was yielded after pooling extracted from three RCTs [16, 17, 19]. Further, the analyses using fixed effect models demonstrated similar results (Table 2 and S2 Fig).

**Table 2:** Summary of results yielded under fixed-effect and random-effects models.

Outcomes	No. of trials	Fixed-effect model			Random-effects model			Heterogeneity	
		Effect size	95% CI	*P* value	Effect size	95% CI	*P* value	*P* value	*I*^*2*^
DGF	6	0.84	0.60–1.18	0.32	0.89	0.61–1.28	0.52	0.40	3%
AR	4	0.95	0.62–1.46	0.81	0.96	0.62–1.49	0.86	0.51	0%
Graft loss	4	0.89	0.35–2.27	0.81	0.90	0.32–2.52	0.84	0.40	0%
50% fall in SCr	3	1.05	0.84–1.30	0.68	1.05	0.70–1.57	0.81	0.03	70%
eGFR 3m	4	1.23	-1.73–4.19	0.41	1.23	-1.73–4.19	0.41	0.61	0%
eGFR 12m	2	3.35	-1.49–8.19	0.18	3.01	-3.26–9.29	0.35	0.21	37%
Hospital stay	4	-0.73	-1.56–0.11	0.09	-0.73	-1.56–0.11	0.09	0.46	0%

DGF: delayed graft function; AR: acute rejection; SCr: serum creatinine; eGFR: estimated glomerular filtration rate; CI: confidence interval.

#### eGFR at 3 months and 12 months

**Fig 4:**
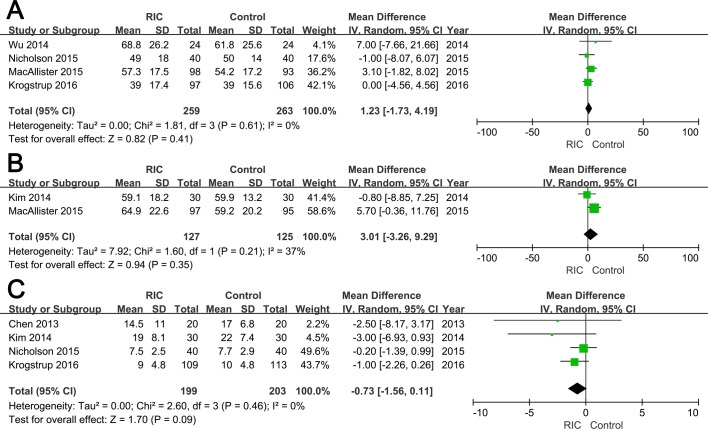
Forest plot with 95% confidence interval for continuous secondary end points (random-effects model). The eGFR at three months post operation (A), eGFR at 12 months post transplantation (B), and hospital stay (C) in recipients treated with RIC compared with controls.

Data regarding eGFR at 3 months and 12 months were reported in four [16–18, 20] and two RCTs [17, 19], respectively. No significant difference was observed between two groups neither at 3 months (RIC vs. Control group: WMD = 1.23; 95% CI, -1.73–4.19; *P* = 0.41; **Fig 4A**) nor at 12 months (WMD = 3.01; 95% CI, -3.26–9.29; *P* = 0.35; **Fig 4B**)., which were similar with the results under fixed effect model (Table 2 and S3 Fig)

#### Hospital stay

Among six included studies, four reported mean length of hospital stay [15, 16, 19, 20]. There was no statistically significant difference in the hospital stay between two groups (WMD = -0.73; 95% CI, -1.56–0.11; *P* = 0.09; **Fig 4C**)

### Heterogeneity evaluation and sensitivity analysis

In the current meta-analysis, significant heterogeneity was only observed in the pooled result of the incidence of 50% fall in SCr. Additionally, sensitivity analyses were also conducted on all outcomes to detect the potential impact of each individual study on the pooled results. The results indicated that one trial contributed to the main source of heterogeneity in terms of the incidence of 50% fall in SCr [19]. No other single study delivered substantial power to influence the other pooled outcomes significantly.

## Discussion

In renal transplantation, development of novel therapeutic approaches to protect renal allografts against IRI is a never-ending challenge, and RIC has emerged as a potent renoprotective strategy [26]. To the best of our knowledge, this is the first meta-analysis to comprehensively evaluate the clinical efficacy and safety of RIC in human kidney transplantation. The results demonstrated that RIC exhibited similar outcomes when compared with the control group, in terms of all primary and secondary endpoints.

RIC was initially performed by Przyklenk et al, and proven to protect myocardium from subsequent IRI induced by sustained coronary occlusion [27]. The improved renal function induced by RIC was initially reported in 2007 by Ali and colleagues in patients treated with elective abdominal aortic aneurysm repair [28], and further validated by Soendergaard and coworkers in a porcine kidney transplantation model [29].

Encouraged by the positive results of RCTs conducted in cardiovascular surgeries and pre-clinical studies in large animal transplantation models, Chen and colleagues conducted the first RCT to explore the clinical efficacy of RIPrC in human renal transplantation. However, unfortunately, the results failed to draw a positive conclusion [15]. Subsequently, another five RCTs were conducted with inconsistent results, and in most of these five studies, RIPoC or RIPeC rather than RIPrC were applied, which could be explained partially by their relatively higher practicability in the clinical setting [16–20]. Recently, Zhang et al conducted a meta-analysis by pooling 37 RCTs with 8168 patients, and proved that RIC might be beneficial for the protection of renal function in different surgeries [30]; however, only one study involved renal transplantation [18].

In the present meta-analysis, with data from six included RCTs, we demonstrated that RIC administration could decrease the DGF rate (random-effects model: RR = 0.89; fixed-effect model: RR = 0.84); however, this result showed no statistical significance. Moreover, similar results were also generated in terms of the incidence of AR, 50% fall of SCr, graft loss, as well as the continuous variables including eGFR and hospital stay.

Differed from the pre-clinical studies with encouraging results [29], both RCTs and this meta-analysis did not demonstrate positive results, which may be attributed to the following aspects: 1) donor type. The RCTs included in our study are mostly living donor kidney transplantation [15–17, 19]. Unlike renal allografts retrieved from deceased donors, those from living donors are subjected to relatively mild IRI, which might hinder the full realization of RIC’s renoprotective effects[13, 18]. 2) renal replacement therapy (RRT). Due to the sustained organ shortage, most ESRD patients cannot receive renal transplantation immediately, and RRT acts as one indispensable therapy during their waiting time. Evidence from the included RCTs revealed that 83.8% of recipients have got preoperative RRT, among which 60.2% were hemodialysis. Mounting evidence indicated that hemodialysis could render repetitive episodes of ischemia and reperfusion, which might induce conditioning effects and suppress any following conditioning interventions including RIC [31, 32]. 3) number of participants. The sample size of each individual study (ranged from 40 to 222) was relatively small. Based on the DGF rate calculated in this meta-analysis (RIC group: 14.2%, Control group: 17.2%; relative risk reduction: 17.5%), a total of 4637 recipients are needed to draw a relatively stable statistical significant result in terms of DGF incidence. 4) inherent drawbacks of RCT. Unlike standardized animal experiments, various confounding factors such as human leukocyte antigen mismatch and administration of immunosuppressive drugs are involved in clinical trials, which may influence the severity of IRI and efficacy of RIC.

Several limitations of this study should be acknowledged when interpreting the results. First, the relatively small sample size of included trials could decrease the statistical power. Second, varied definitions of DGF and AR were applied in these RCTs, which could impact on the combined results directly. Third, the follow-up time ranged from 3 months to 12 months among the six RCTs, which limited the assessment of long-term renal function. Lastly, due to the methodological limitations, this meta-analysis could conclude false negative results, and the clinical efficacy of RIC in renal transplantation should be validated in further well-designed RCTs with large sample size.

## Conclusions

In summary, the present meta-analysis suggested that RIC might exert renoprotective functions in human kidney transplantation. However, the clinical utility remained limited, and further well-designed RCTs with large sample size are needed to assess this clinical efficacy and safety of RIC in renal transplantation.

## Supporting Information

S1 PRISMA Checklist PRISMA Checklist for this meta-analysis. (DOC)

S1 Table Full electronic search strategy in PubMed. (DOCX)

S2 Table Raw data extracted from six randomized controlled trials. (XLSX)

S3 Table Quality assessment of included studies. (DOCX)

S1 Fig Forest plot with 95% confidence interval in DGF rates (fixed effect model). Stratification analysis was conducted based on RIC types (RIPrC, RIPoC, and RIPeC).
(TIF)

S2 Fig Forest plot with 95% confidence interval for dichotomous secondary end points (fixed effect model). The incidence of AR (A), graft loss (B), and 50% fall in serum creatinine (C) in recipients treated with RIC compared with controls.
(TIF)

S3 Fig Forest plot with 95% confidence interval for continuous secondary end points (fixed effect model). The eGFR at three months post operation (A), eGFR at 12 months post transplantation (B), and hospital stay (C) in recipients treated with RIC compared with controls.
(TIF)
