# Correction: Limited Clinical Utility of Remote Ischemic Conditioning in Renal Transplantation: A Meta-Analysis of Randomized Controlled Trials

**DOI:** 10.1371/journal.pone.0204184

**Published:** 2018-09-14

**Authors:** Chang-Cheng Zhou, Yu-Zheng Ge, Wen-Tao Yao, Ran Wu, Hui Xin, Tian-Ze Lu, Ming-Hao Li, Kai-Wei Song, Min Wang, Yun-Peng Zhu, Meng Zhu, Li-Guo Geng, Xiao-Fei Gao, Liu-Hua Zhou, Sheng-Li Zhang, Jia-Geng Zhu, Rui-Peng Jia

In the original article [1], the reporting of the inclusion and exclusion criteria for the meta-analysis was incomplete. The meta-analysis included eligible clinical studies for perioperative RIP, namely pre-RIP (RIPrC), per-RIP (RIPeC), and post-RIP (RIPoC). Studies of patients with RIP at 24 h prior surgery (“late RIPC”) were not included, as early RIPC is more practicable than late RIPC in the clinical setting.

The REPAIR Trial, published by MacAllister et al. (2015) [2] included a total of 406 donor–recipient pairs in four groups: sham RIPC (n = 99), early RIPC (immediately before surgery, n = 102), late RIPC (24 hours before surgery, n = 103) and dual RIPC (early and late RIPC, n = 102). In accordance with the above inclusion criteria, the meta-analysis excluded two groups from the REPAIR Trial (late RIPC and dual RIPC). To clarify this, Table 1 is corrected to include an additional footnote as follows:

**Table 1:** Characteristics of included trials.

Author	Year	No. of patients	Age (y)	Males (%)	RIC type	RIC procedure	Donor type	Preoperative HD n(%)
Krogstrup	2016	109/113	58.1(49.5–65.0)/ 61.4(49.4–66.6)	60/61	perconditioning	4 cycles of 5-minute ischemia and 5-minute reperfusion of the thigh	DCD/DBD	68(62)/65(58)
Nicholson	2015	40/40	45±14/47±14	67.5/52.5	perconditioning	4 cycles of 5-minute ischemia and 5-minute reperfusion of the thigh	Living-donor	23(58) / 20(50)
MacAllister^a^	2015	102/99	47.6±15.1/46.8±15.1	72.4/61.1	preconditioning	4 cycles of 5-minute ischemia and 5-minute reperfusion of the arm	Living-donor	51(50) / 51(52)
Wu	2014	24/24	40.6±11.6/39.7±10.2	50/62.5	perconditioning	3 cycles of 5-minute ischemia and 5-minute reperfusion of the iliac artery	DCD	18(75) / 17(71)
Kim	2014	30/30	49(39–52)/46(36–50)	66.7/70	postconditioning	3 cycles of 5-minute ischemia and 5-minute reperfusion of the arm	Living-donor	27(90) / 28(93)
Chen	2013	20/20	30.6±7.0/32.5±10.3	70/80	preconditioning	3 cycles of 5-minute ischemia and 5-minute reperfusion of the thigh	Living-donor	Unclear

DCD: donation after cardiac death; DBD: donation after brain death; HD: hemodialysis; RIC: remote ischemic conditioning.

^a^ Note that for the MacAllister 2015 trial, data from the following two groups of the trial were excluded from this meta-analysis and are not presented in the table: late RIPC (24 hours before surgery, n = 103) and dual RIPC (24 hours before and immediately before surgery, n = 102).

Partial data from the study by MacAllister et al. (2015) was incorrectly extracted, which augmented the number of events including delayed graft function (DGF), acute rejection (AR), graft loss, and 50% fall in SCr. The authors have carefully re-extracted all the four inappropriate outcomes data and re-analyzed all involved results in the meta-analysis. The details of the modifications are listed below.

The incidence of DGF is set as the primary endpoint in the study. After reanalysis with the re-extracted data, there are some modifications in the text of the Abstract, Discussion, and Results sections with corrections shown here:

In the Abstract and Discussion sections, “random-effects model: RR = 0.89; fixed-effect model: RR = 0.84” is corrected to “random-effects model: RR = 0.86; fixed-effect model: RR = 0.81”.

The correct sentence regarding the incidence of DGF in the “Study outcomes” section of the Results is: “After pooling data from all six trials, a trend of decline in the risk of DGF could be observed in the RIC group than the control group both under random-effects (RR = 0.86; Fig 2) and fixed-effect models (RR = 0.81; S1 Fig). However, unfortunately, the decrease did not demonstrate statistical significance under both models (random-effects model: 95% CI, 0.61–1.22, P = 0.41; fixed effect model: 95% CI, 0.57–1.13, P = 0.22;)”.

**Fig 2:**
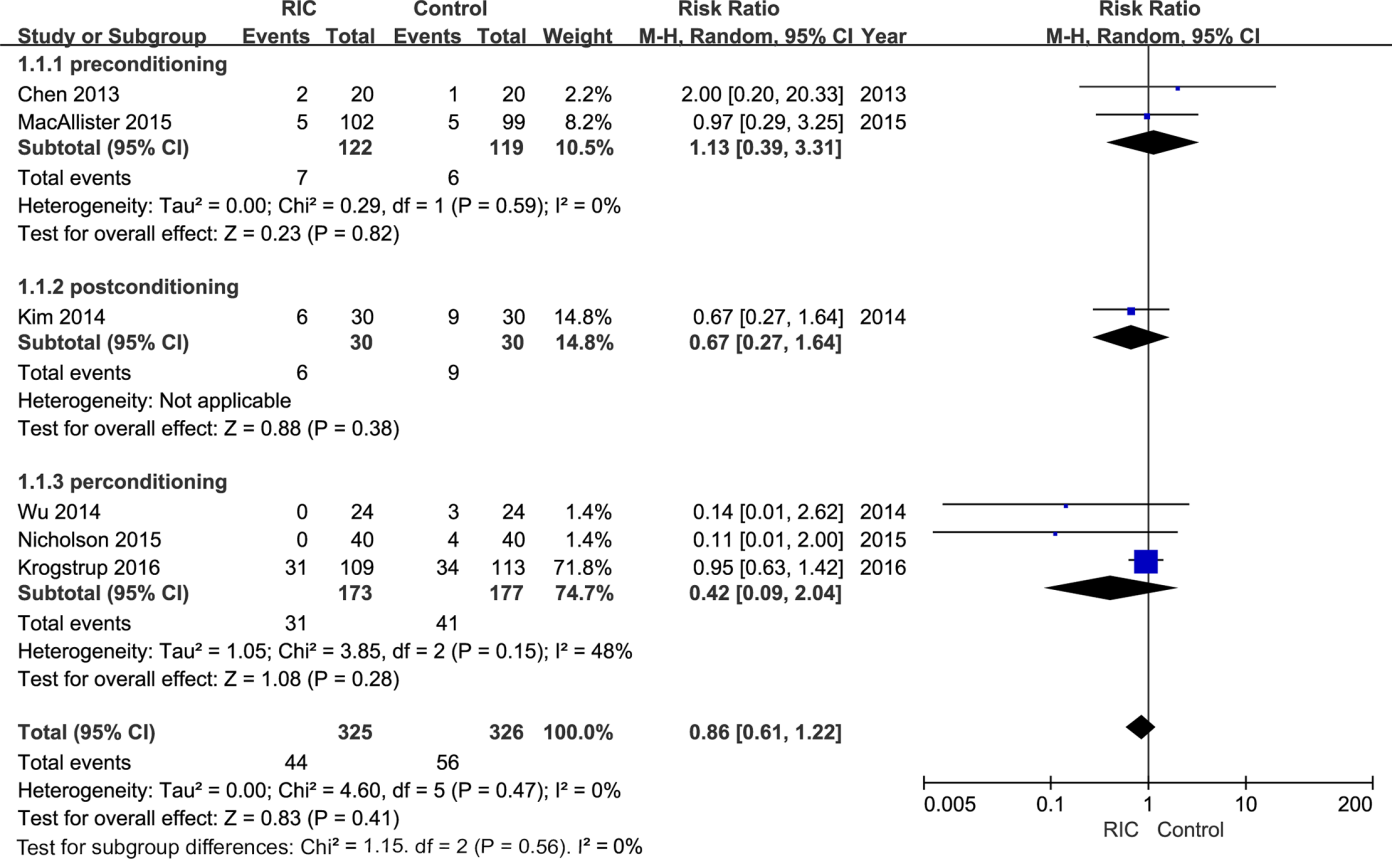
Forest plot with 95% confidence interval in DGF rates (random-effects model). Stratification analysis was conducted based on RIC types (RIPrC, RIPoC, and RIPeC).

In the fifth paragraph of the Discussion section, the needed sample size was also calculated based on the DGF rate. Thus, the revised sentence is: “Based on the DGF rate calculated in this meta-analysis (RIC group: 13.5%, Control group: 17.2%; relative risk reduction: 21.5%), a total of 2980 recipients are needed to draw a relatively stable statistical significant result in terms of DGF incidence”.

Moreover, the secondary endpoints including the incidence of AR, graft loss and 50% fall in SCr are also modified. The correct sentence in the Results section entitled “Incidence of AR, graft loss, 50% fall in SCr” is: “No significant difference was detected in the incidence of AR between two groups (RIC vs. Control groups: RR = 0.87; 95% CI, 0.47–1.61; P = 0.65; Fig 3A). The incidence of graft loss within three months was documented in four studies, and no significant difference was observed between groups (RIC vs. Control groups: RR = 1.36; 95% CI, 0.45–4.10; P = 0.58; Fig 3B). With regard to the rate of 50% fall in SCr within 24h post-transplantation, no statistical significant data (RIC vs. Control groups: RR = 1.01; 95% CI, 0.61–1.66; P = 0.98; Fig 3C) was yielded after pooling extracted from three RCTs”.

**Fig 3:**
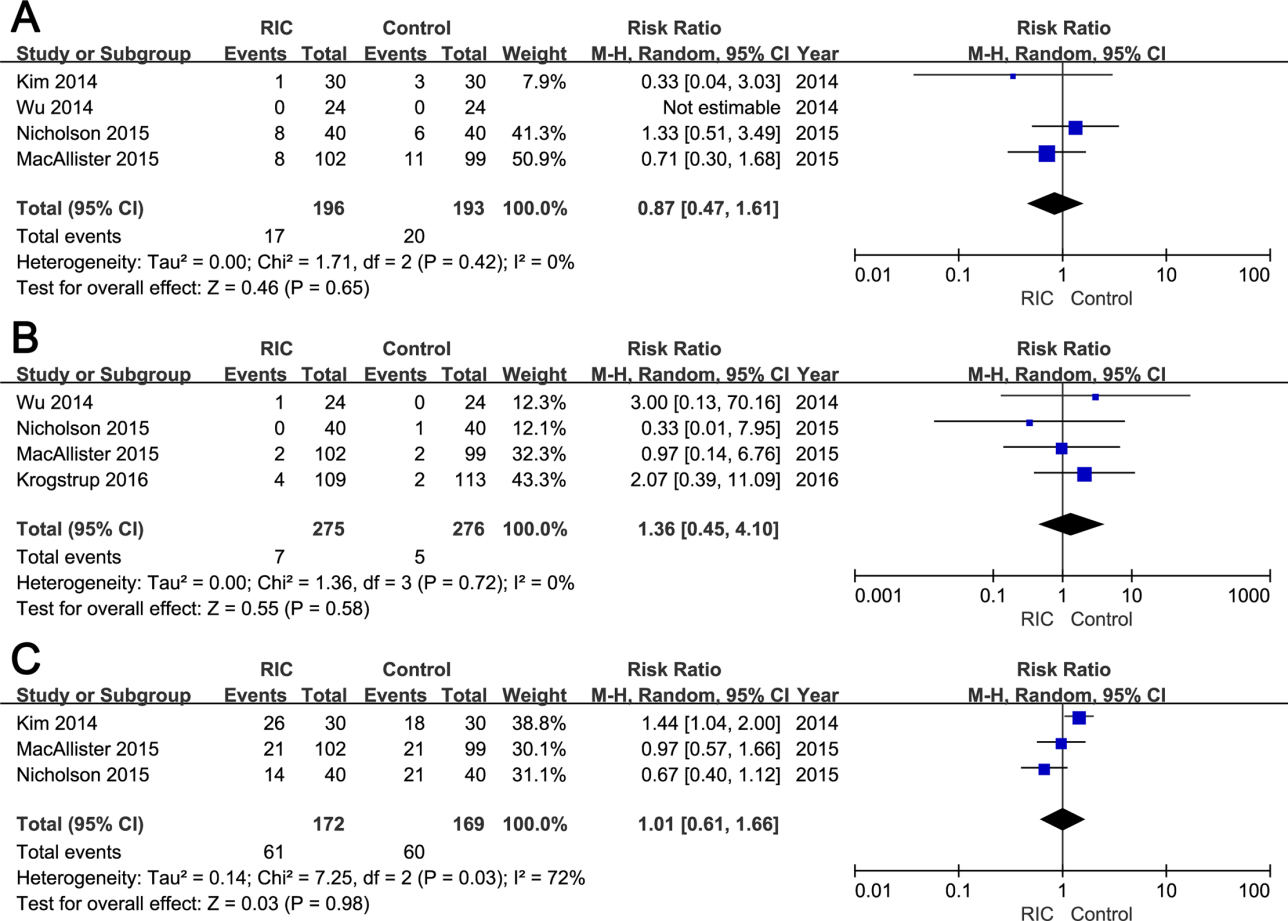
Forest plot with 95% confidence interval for dichotomous secondary end points (random-effects model). The incidence of AR (A), graft loss (B), and 50% fall in serum creatinine (C) in recipients treated with RIC compared with controls.

The Results text in the section “Hospital Stay” is also modified. The correct sentence is: “Among six included studies, four reported mean length of hospital stay [15, 16, 19, 20]. There was no statistically significant difference in the hospital stay between two groups (WMD = -0.70; 95% CI, -1.53–0.14; *P* = 0.10; Fig 4C)”.

Please see the corrected Figs 2 and 3 (random-effects models) and Table 2 below. The corrected versions of S2 Table (raw data) and S1–S3 Figs (fixed-effect models) are included in the Supporting Information section of this notice. Additionally, Fig 4 has been modified to be more exact and readable; the corrected Fig 4 has also been included below.

**Fig 4:**
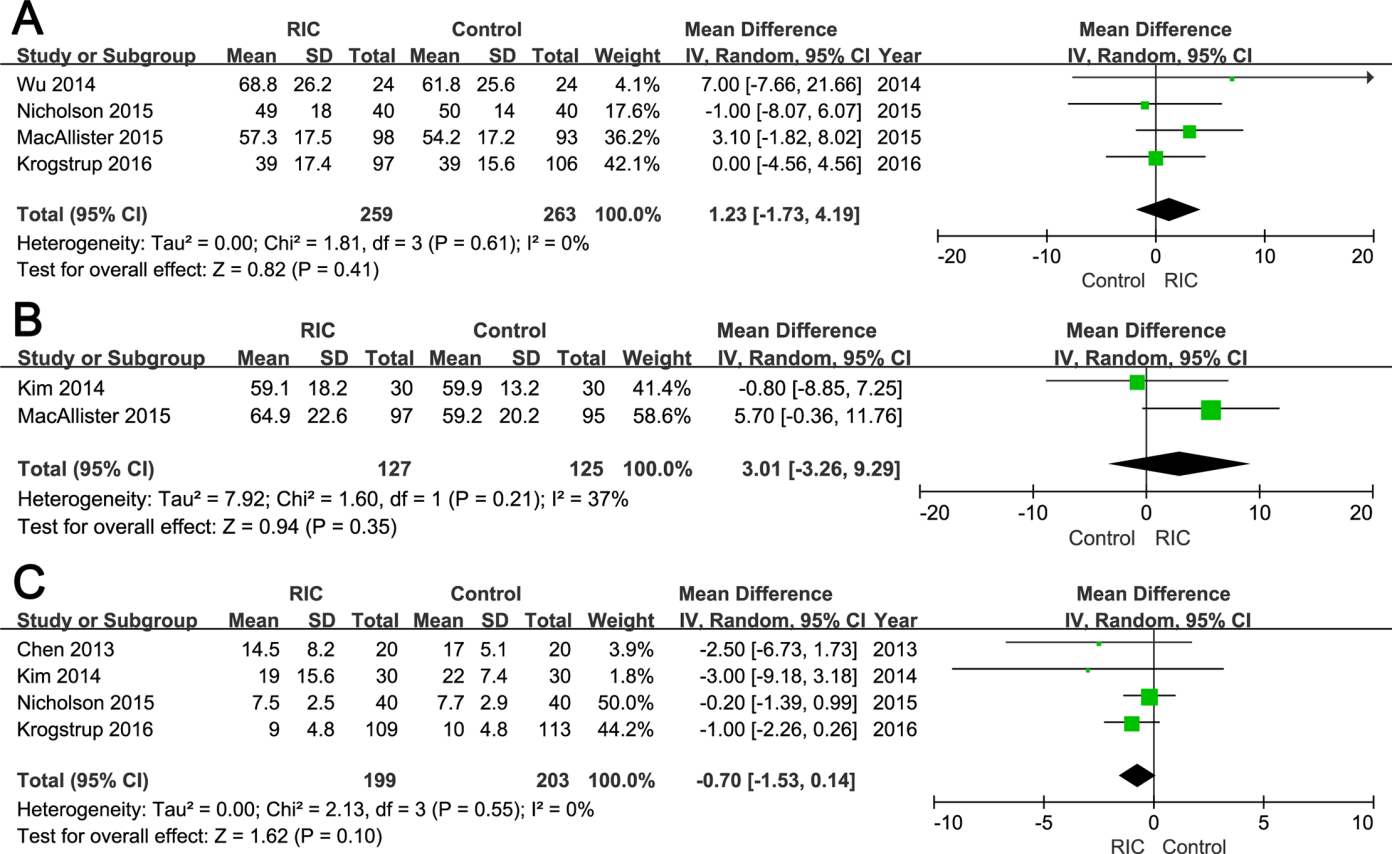
Forest plot with 95% confidence interval for continuous secondary end points (random-effects model). The eGFR at three months post operation (A), eGFR at 12 months post transplantation (B), and hospital stay (C) in recipients treated with RIC compared with controls.

**Table 2:** Summary of results yielded under fixed-effect and random-effects models.

Outcomes	No. of trials	Fixed-effect model			Random-effects model			Heterogeneity	
		Effect size	95% CI	*P* value	Effect size	95% CI	*P* value	*P* value	*I*^*2*^
DGF	6	0.81	0.57–1.13	0.22	0.86	0.61–1.22	0.41	0.47	0%
AR	4	0.84	0.46–1.54	0.57	0.87	0.47–1.61	0.65	0.42	0%
Graft loss	4	1.34	0.47–3.81	0.58	1.36	0.45–4.10	0.58	0.72	0%
50% fall in SCr	3	1.01	0.77–1.31	0.96	1.01	0.61–1.66	0.98	0.03	72%
eGFR 3m	4	1.23	-1.73–4.19	0.41	1.23	-1.73–4.19	0.41	0.61	0%
eGFR 12m	2	3.35	-1.49–8.19	0.18	3.01	-3.26–9.29	0.35	0.21	37%
Hospital stay	4	-0.70	-1.53–0.14	0.10	-0.70	-1.53–0.14	0.10	0.55	0%

DGF: delayed graft function; AR: acute rejection; SCr: serum creatinine; eGFR: estimated glomerular filtration rate; CI: confidence interval.

It is important to note that the above changes do not affect the overall conclusions of the original article.

To elucidate the conclusions of the study more accurately, the conclusions are reiterated as follows: In summary, the present meta-analysis suggests that perioperative pre-RIP, per-RIP or post-RIP procedures showed no statistically significant protective impact on kidney transplant functions. Consequently, the clinical utility of RIP remains limited. Further well-designed RCTs with large sample size would need to be performed to document the efficacy and safety of RIC in renal transplantation.

With regard to the methodological information of the article, the following should be clarified:

1) To avoid a possible effect of heterogeneity, the authors had also conducted a subgroup analysis based on different conditioning types, and the results remain unchanged; 2) If continuous data are presented as median and interquartile range (IQR), median was utilized to estimate mean when the sample size is larger than 25 [3], and interquartile range (IQR)/1.35 was treated as an estimator for standard deviation [4]; 3) In this meta-analysis both random-effects models and fixed-effect models were adopted. The authors mainly presented the results yielded under random effects model in the main text, and in the conclusions, while the fixed effect model was used as sensitivity analysis solely. Though the comparison could not be measurable, obvious differences between the two models could not be observed; 4) In the original article, one double-zero study (i.e., no AR events in both RIC and control patients) could be detected when the incidence of AR was evaluated, and the information from this study was not included in the summary effect for AR. The authors re-analyzed the outcomes of AR using SAS (Version 9.2) PROC NLMIXED with a beta-binomial model (the codes were obtained from supplementary of [5]). This new model yielded similar results compared with the old model utilized in the original article (new: RR = 0.87, 95%CI, 0.27–2.79, P = 0.82; old: RR = 0.87, 95%CI, 0.47–1.61, P = 0.65). Furthermore, the sample size of the above double-zero study is also small. Thus, the original synthetic outcomes of AR could be acceptable after excluding the double-zero study; 5) The authors did not have a registered study protocol in a publicly accessible database.

A difference in DGF incidence could be observed between studies. This could be attributed to the following reasons: 1) Different definition of DGF; 2) Different study characteristics; and 3) Different renal allograft source: DBD, DCD or living donor.

## Supporting information

S2 Table Raw data extracted from six randomized controlled trials. (XLSX)

S1 Fig Forest plot with 95% confidence interval in DGF rates (fixed effect model). Stratification analysis was conducted based on RIC types (RIPrC, RIPoC, and RIPeC).
(TIF)

S2 Fig Forest plot with 95% confidence interval for dichotomous secondary end points (fixed effect model). The incidence of AR (A), graft loss (B), and 50% fall in serum creatinine (C) in recipients treated with RIC compared with controls.
(TIF)

S3 Fig Forest plot with 95% confidence interval for continuous secondary end points (fixed effect model). The eGFR at three months post operation (A), eGFR at 12 months post transplantation (B), and hospital stay (C) in recipients treated with RIC compared with controls.
(TIF)
