# Supplementary material for: Limited Clinical Utility of Remote Ischemic Conditioning in Renal Transplantation: A Meta-Analysis of Randomized Controlled Trials
Source: PLoS One. 2017 Jan 27;12(1):e0170729. doi: 10.1371/journal.pone.0170729 (PMC5271340; doi:10.1371/journal.pone.0170729)
Supplement: S1 Table — (DOCX) [file pone.0170729.s002.docx]

S1 Table. Full electronic search strategy in PubMed

| **Database** | **Search Strategy** |
| --- | --- |
| **PubMed** | ((kidney transplant) OR renal transplant) AND ((((ischemic preconditioning) OR ischemic postconditioning) OR ischemic conditioning) OR ischemic perconditioning) |
