# Supplementary material for: Limited Clinical Utility of Remote Ischemic Conditioning in Renal Transplantation: A Meta-Analysis of Randomized Controlled Trials
Source: PLoS One. 2017 Jan 27;12(1):e0170729. doi: 10.1371/journal.pone.0170729 (PMC5271340; doi:10.1371/journal.pone.0170729)
Supplement: S3 Table — (DOCX) [file pone.0170729.s004.docx]

**S3 Table. Quality assessment of included studies**

| **Author** | **Year** | **Randomization method** | **Blind** | **Explanation for withdrawals/dropouts** | **Jadad Score** | **Allocation concealment** | **Intention-to-treat analysis** |
| --- | --- | --- | --- | --- | --- | --- | --- |
| Krogstrup | 2016 | Online randomisation programme | Double | YES | 5 | Unclear | YES |
| Nicholson | 2015 | Computer generated | Double | YES | 5 | Sealed envelopes | YES |
| MacAllister | 2015 | Ceb-based service | Double | YES | 5 | Sealed envelopes | YES |
| Wu | 2014 | Unclear | Single | YES | 2 | Unclear | Unclear |
| Kim | 2014 | Computer generated | Double | YES | 5 | Sealed envelopes | Unclear |
| Chen | 2013 | Unclear | Single | YES | 2 | Unclear | Unclear |
