# Supplementary figures and images for: Limited Clinical Utility of Remote Ischemic Conditioning in Renal Transplantation: A Meta-Analysis of Randomized Controlled Trials
Source: PLoS One. 2017 Jan 27;12(1):e0170729. doi: 10.1371/journal.pone.0170729 (PMC5271340; doi:10.1371/journal.pone.0170729)

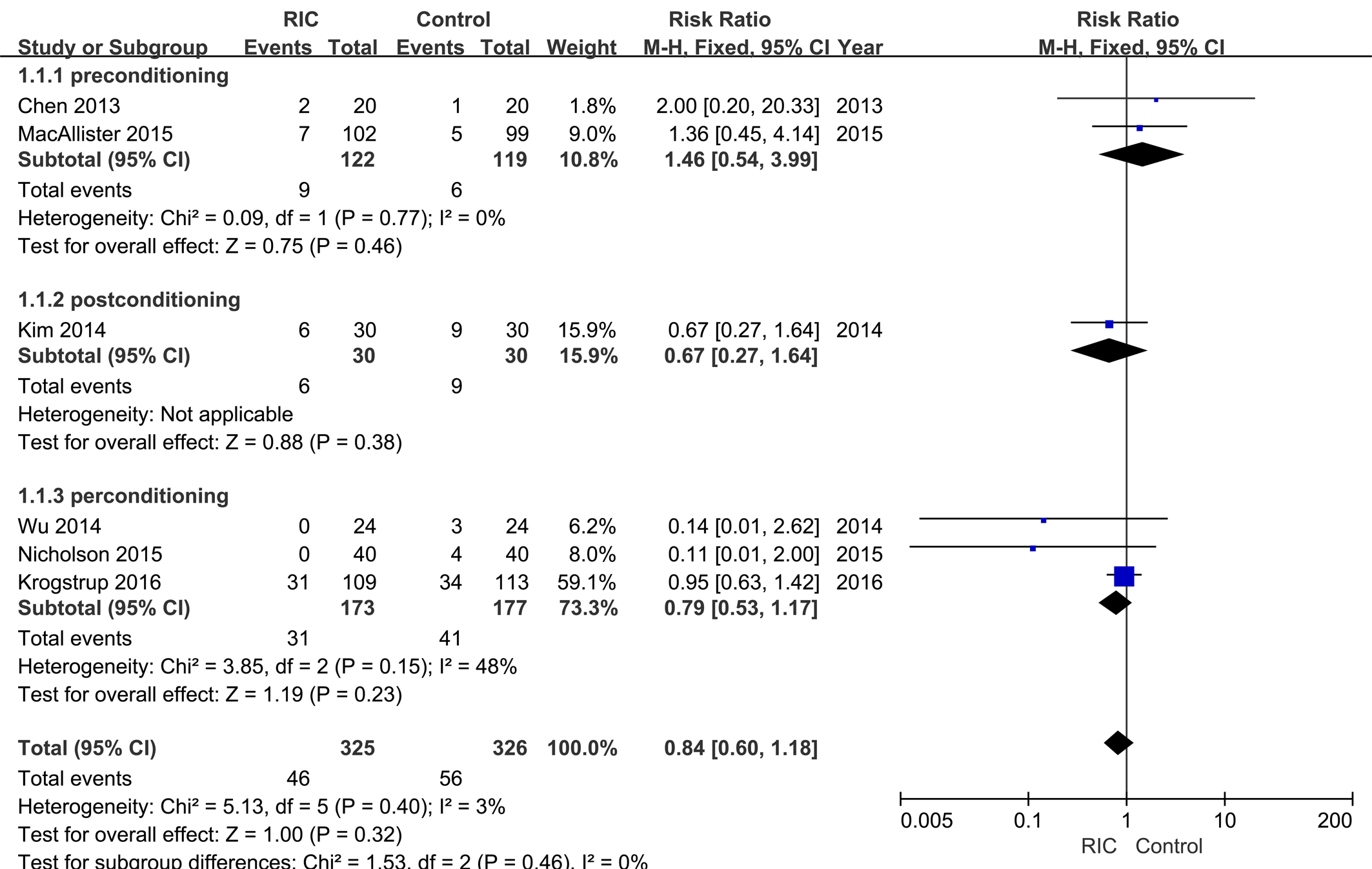

Supplement: S1 Fig — Stratification analysis was conducted based on RIC types (RIPrC, RIPoC, and RIPeC). (TIF) [file pone.0170729.s005.tif]

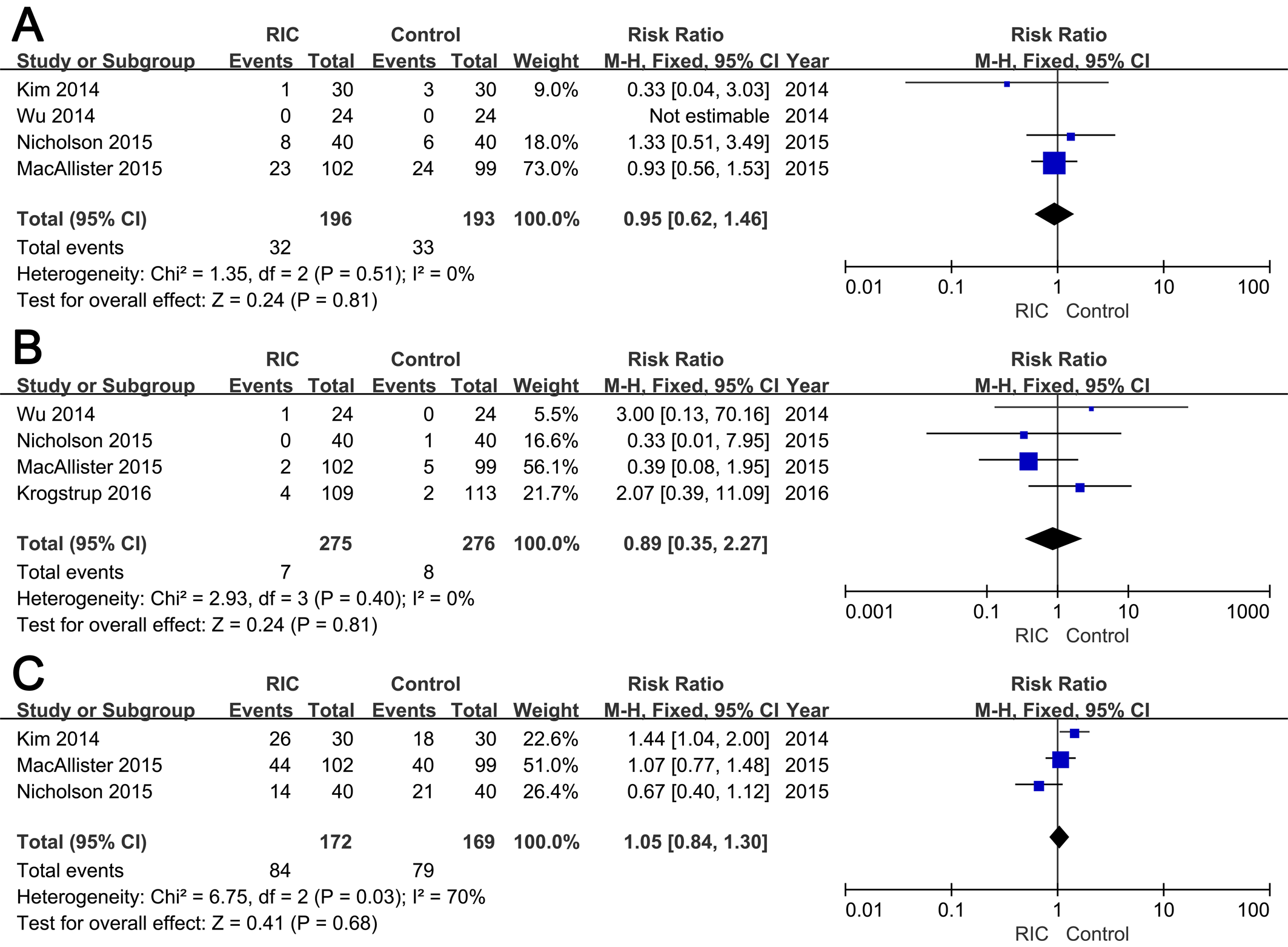

Supplement: S2 Fig — The incidence of AR (A), graft loss (B), and 50% fall in serum creatinine (C) in recipients treated with RIC compared with controls. (TIF) [file pone.0170729.s006.tif]

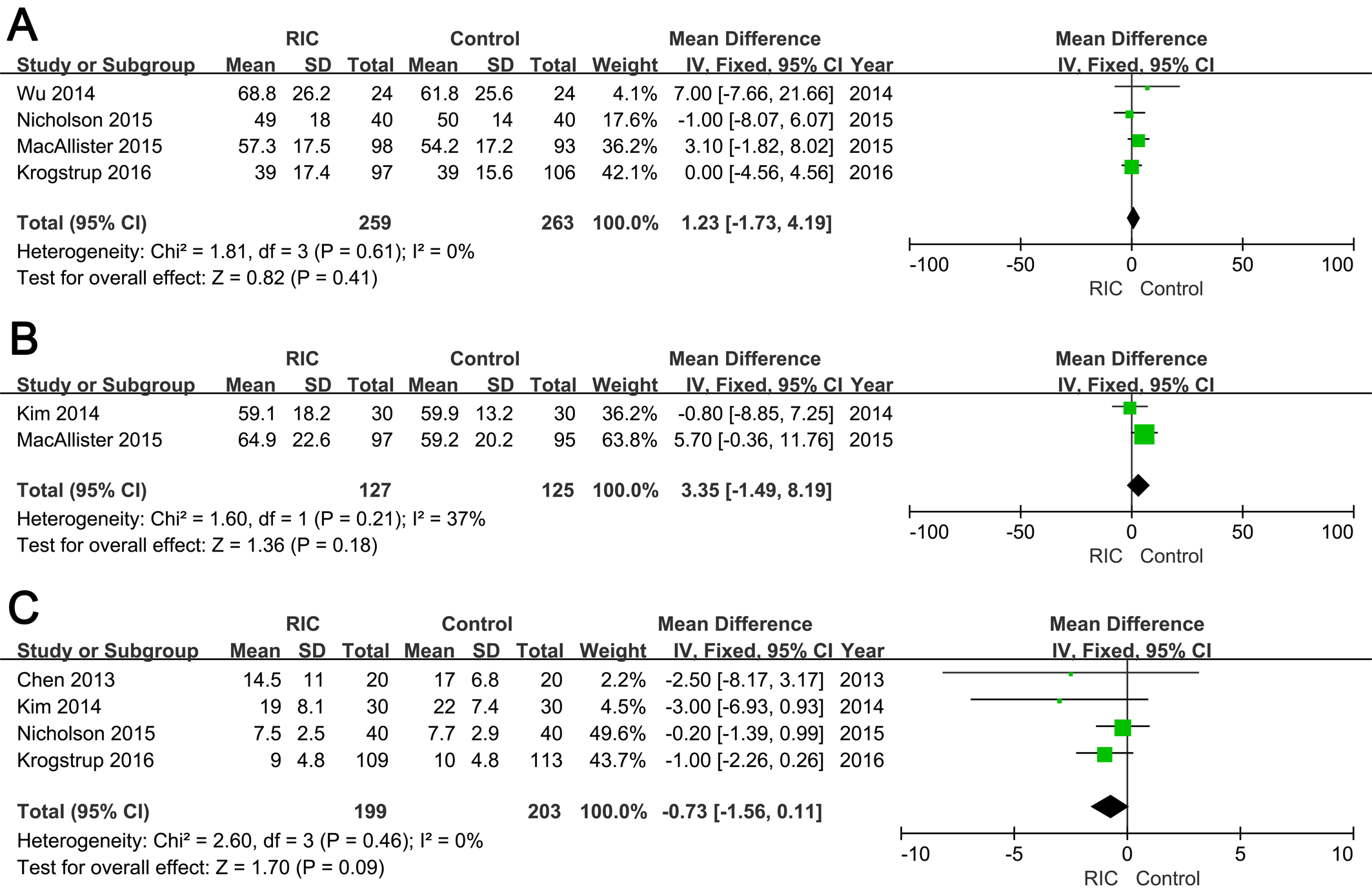

Supplement: S3 Fig — The eGFR at three months post operation (A), eGFR at 12 months post transplantation (B), and hospital stay (C) in recipients treated with RIC compared with controls. (TIF) [file pone.0170729.s007.tif]
