# Supplementary figures and images for: Correction: Limited Clinical Utility of Remote Ischemic Conditioning in Renal Transplantation: A Meta-Analysis of Randomized Controlled Trials
Source: PLoS One. 2018 Sep 14;13(9):e0204184. doi: 10.1371/journal.pone.0204184 (PMC6138457; doi:10.1371/journal.pone.0204184)

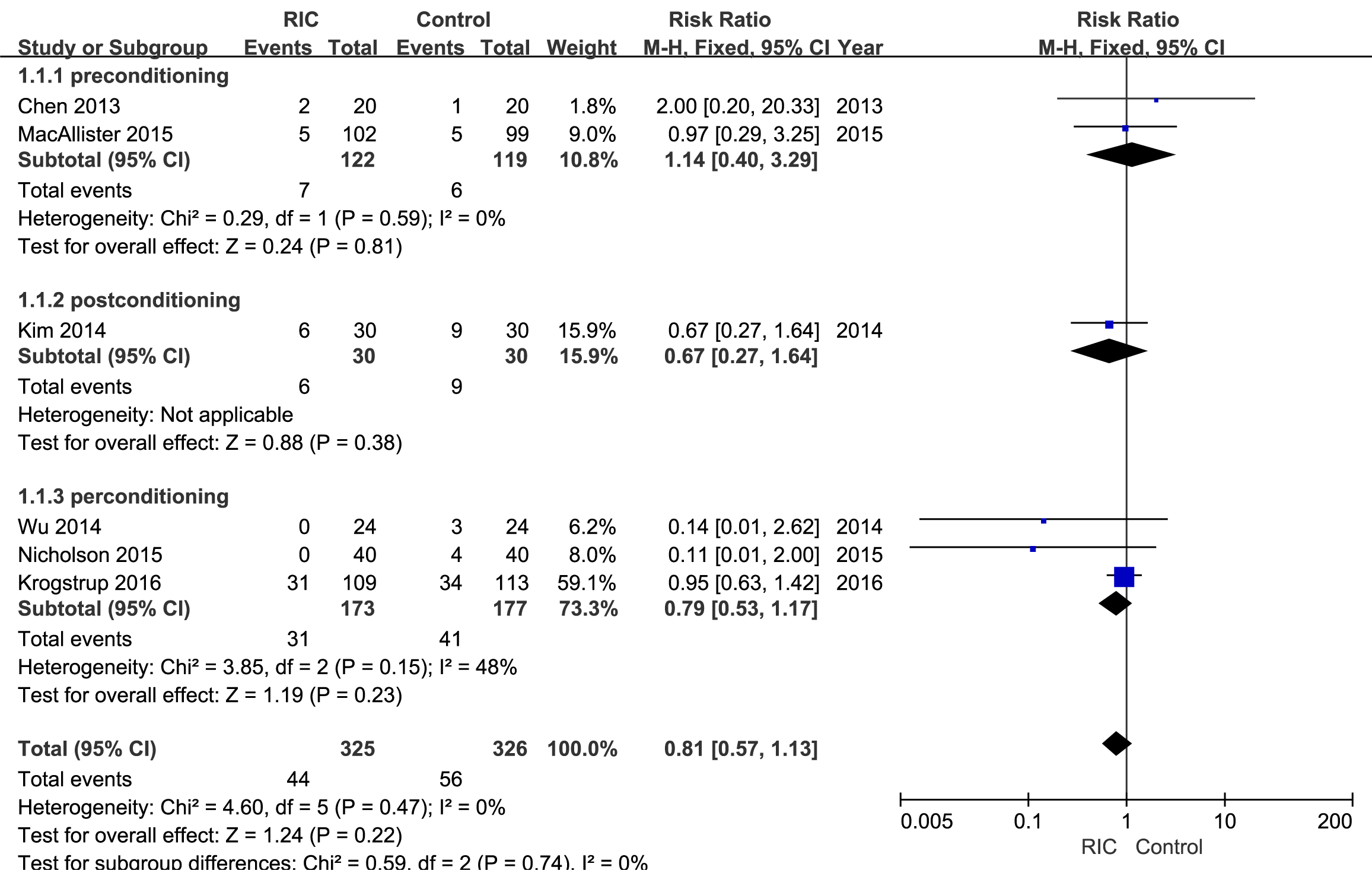

Supplement: S1 Fig — Stratification analysis was conducted based on RIC types (RIPrC, RIPoC, and RIPeC). (TIF) [file pone.0204184.s002.tif]

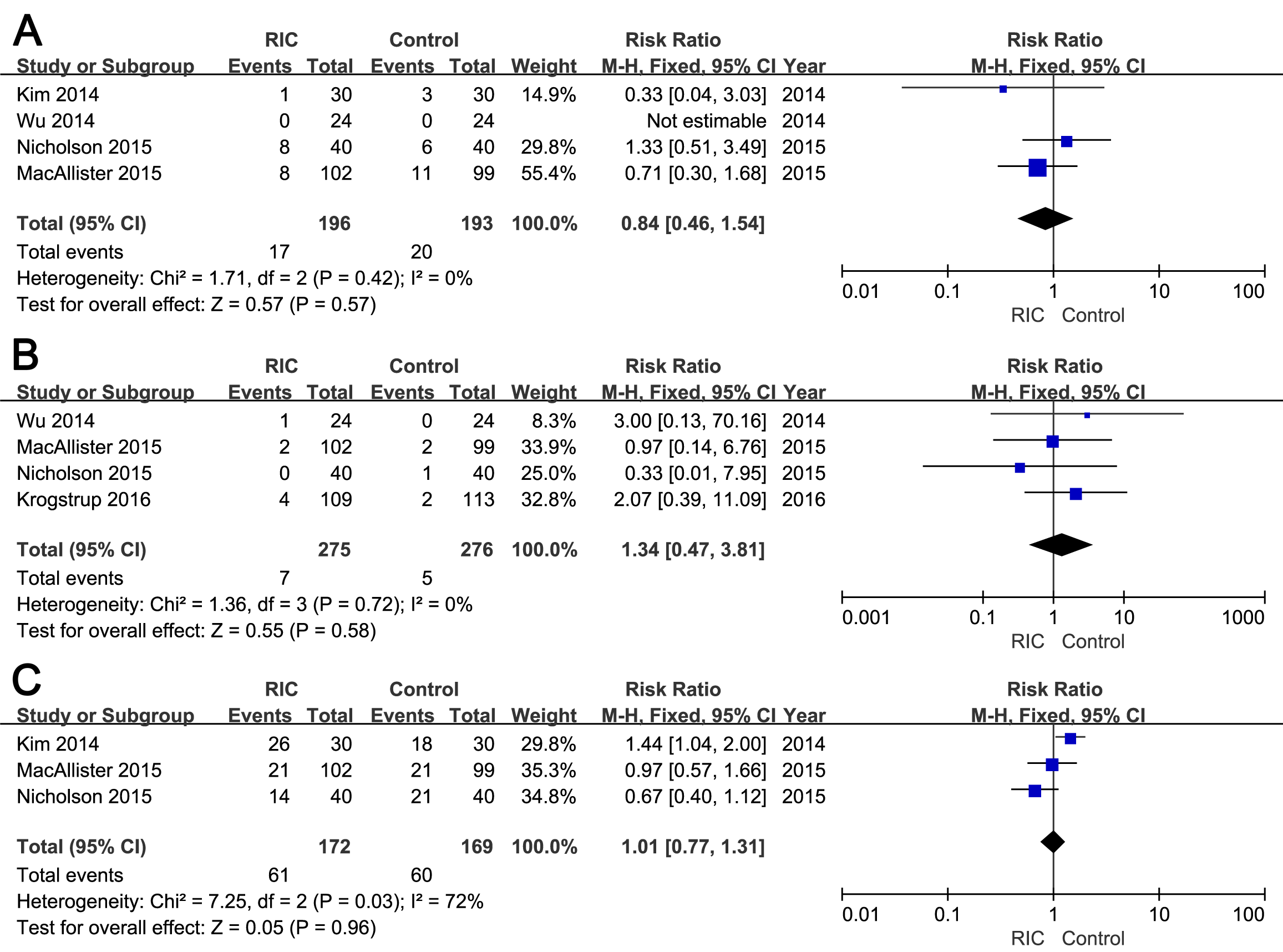

Supplement: S2 Fig — The incidence of AR (A), graft loss (B), and 50% fall in serum creatinine (C) in recipients treated with RIC compared with controls. (TIF) [file pone.0204184.s003.tif]

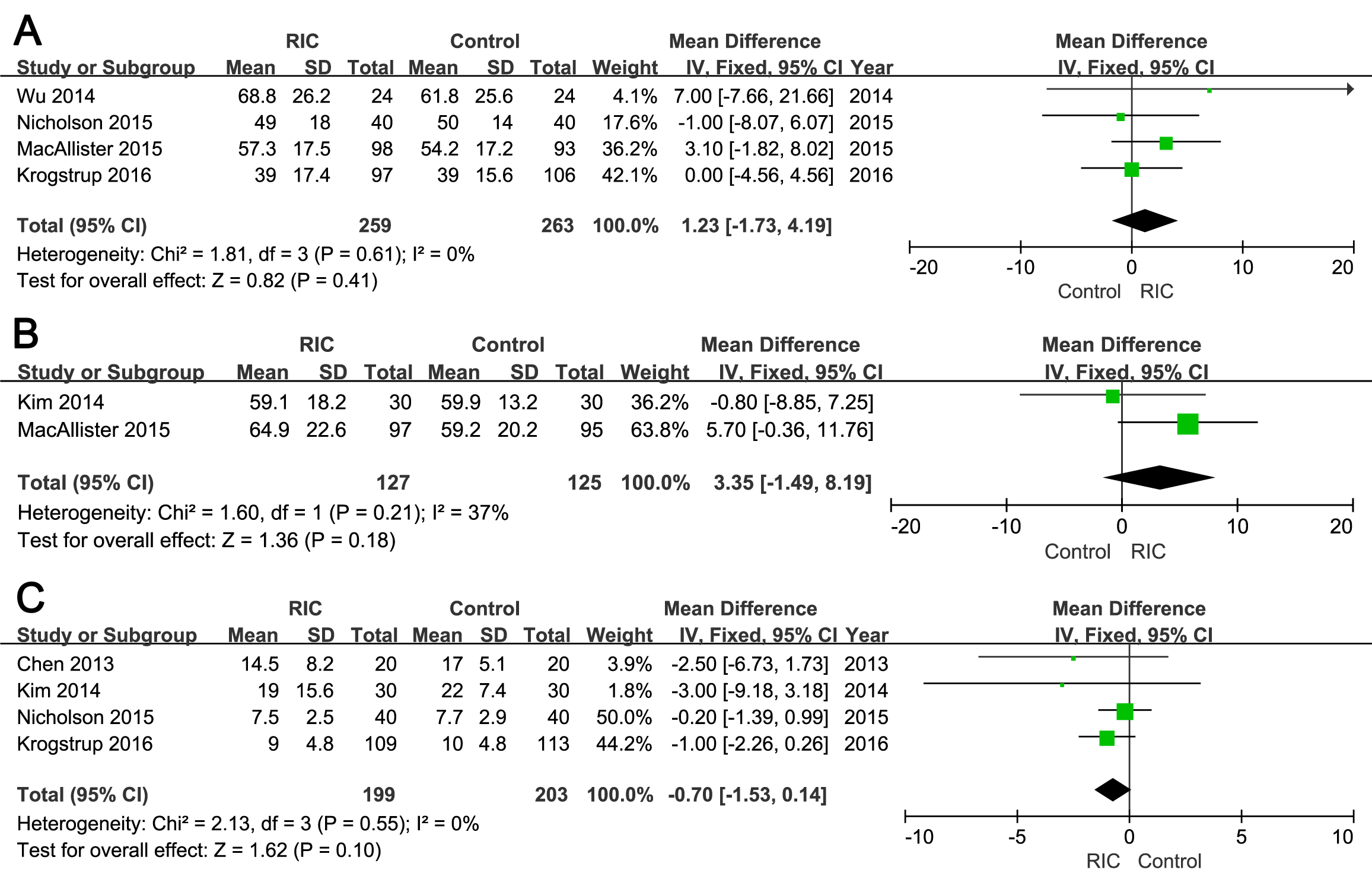

Supplement: S3 Fig — The eGFR at three months post operation (A), eGFR at 12 months post transplantation (B), and hospital stay (C) in recipients treated with RIC compared with controls. (TIF) [file pone.0204184.s004.tif]
